# Optimization of avian perching manoeuvres

**DOI:** 10.1038/s41586-022-04861-4

**Published:** 2022-06-29

**Authors:** Marco KleinHeerenbrink, Lydia A. France, Caroline H. Brighton, Graham K. Taylor

**Affiliations:** 1 Department of Zoology, University of Oxford, Oxford, UK; 2 Present Address: The Alan Turing Institute, London, UK

**Keywords:** Animal behaviour, Biomechanics, Aerospace engineering

## Abstract

Perching at speed is among the most demanding flight behaviours that birds perform^1,2^ and is beyond the capability of most autonomous vehicles. Smaller birds may touch down by hovering^3–8^, but larger birds typically swoop up to perch^1,2^—presumably because the adverse scaling of their power margin prohibits hovering^9^ and because swooping upwards transfers kinetic to potential energy before collision^1,2,10^. Perching demands precise control of velocity and pose^11–14^, particularly in larger birds for which scale effects make collisions especially hazardous^6,15^. However, whereas cruising behaviours such as migration and commuting typically minimize the cost of transport or time of flight^16^, the optimization of such unsteady flight manoeuvres remains largely unexplored^7,17^. Here we show that the swooping trajectories of perching Harris’ hawks (*Parabuteo unicinctus*) minimize neither time nor energy alone, but rather minimize the distance flown after stalling. By combining motion capture data from 1,576 flights with flight dynamics modelling, we find that the birds’ choice of where to transition from powered dive to unpowered climb minimizes the distance over which high lift coefficients are required. Time and energy are therefore invested to provide the control authority needed to glide safely to the perch, rather than being minimized directly as in technical implementations of autonomous perching under nonlinear feedback control^12^ and deep reinforcement learning^18,19^. Naive birds learn this behaviour on the fly, so our findings suggest a heuristic principle that could guide reinforcement learning of autonomous perching.

## Abstract (web summary)

To perch safely, large birds minimize the distance flown after stalling when swooping up from a dive to a perch, but not the time or energy required.

## Main

The exquisite perching performance of birds has inspired many efforts to achieve similar capabilities in autonomous aircraft^10,12,13,18,20–24^. Perching is made demanding by the lack of a runway to bleed speed after landing, which creates a precise targeting requirement that is exacerbated by the difficulty of maintaining control authority at the low airspeeds needed before touchdown^10,12,20^. Although some kinetic energy is converted to gravitational potential energy when climbing to perch^1,2,10^, most is either lost through aerodynamic drag or dissipated on impact^3,7,25–27^. Powerful aerodynamic braking is therefore key to avoiding a dangerously energetic collision, but the high angles of attack that this requires will compromise control as the wing stalls^10,12,20,24^. Birds delay the onset of stall by executing a characteristic rapid pitch-up manoeuvre when perching^1,2,6,22,24,28^, but the rapidity of this manoeuvre leaves little room for error and makes the optimization of its entry conditions critical^18^. This begs the question of how the perching trajectories of birds are optimized and offers a tractable test case for understanding how animals optimize complex unsteady motions^7^.

## Hawks learn to swoop upwards to a perch

**Fig. 1:**
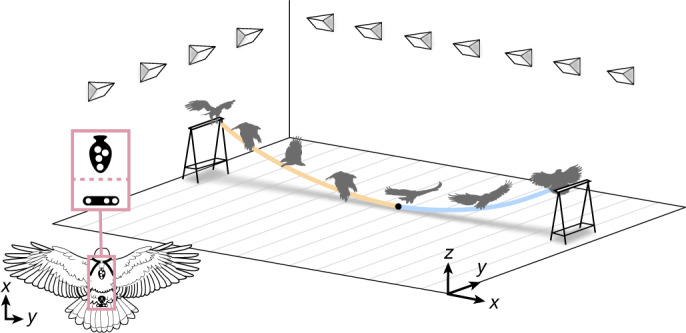
Schematic of a characteristic swooping trajectory and data acquisition. Harris’ hawks were flown between perches in a purpose-built motion capture studio, wearing a template of retroreflective markers close to the centre of mass (inset; tail markers also shown). Swooping was initiated by a take-off jump, followed by a powered dive (yellow line). This transitioned at its lowest point (black dot) into an unpowered climb (blue line), finishing with a rapid pitch-up manoeuvre that ended with the body almost vertical and with the wings outstretched as the feet contacted the perch.

**Fig. 2:**
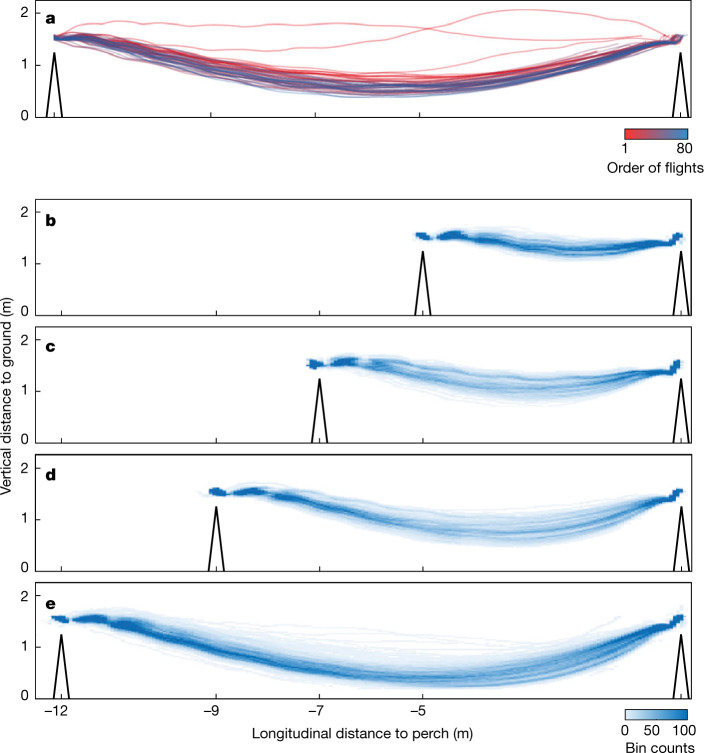
Measured swooping trajectories of perching Harris’ hawks. **a**, Ontogeny of 45 flight trajectories recorded at a perch spacing of 12 m during the initial familiarization period for juvenile bird ‘Toothless’; earlier flights are shown in red, with later flights shown in blue. Note the more direct trajectory taken on earlier flights and quick acquisition of a swooping trajectory characteristic of experienced birds. **b**–**e**, Spatial histograms showing pooled trajectory data from *n* = 1,585 flights for all *n* = 4 hawks at a perch spacing of 5 m (**b**), 7 m (**c**), 9 m (**d**) and 12 m (**e**); see supporting code (10.6084/m9.figshare.16529328). These spatial histograms do not include trajectories recorded during the initial familiarization period.

**Extended Data Fig. 1:**
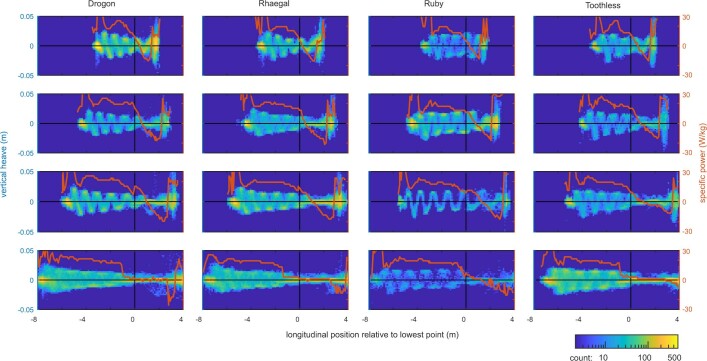
Plot of body heave oscillations removed by lowpass filtering. Two-dimensional histogram of vertical heave oscillations removed by lowpass filtering the body trajectory against longitudinal distance from the lowest point of the filtered trajectory. The orange line shows the associated mean specific power as shown in Fig. 4 of the main text. While some wing motion occurs beyond the observed transition points, the power – and hence the thrust – tends to be substantially reduced.

To address these questions, we rigged a large custom-built motion capture studio to record *n* = 4 captive-bred Harris’ hawks flying between perches for food (Methods and Supplementary Video 1). The hawks wore retroreflective markers enabling us to reconstruct their flight trajectories at a sampling rate of 120 or 200 Hz (Fig. 1). Three of the birds were juvenile males that had only flown short distances previously and thus were initially naive to the task; the other was an experienced adult female. We collected trajectory data from 1,585 flights at perch spacing of 5, 7, 9 or 12 m and perch height of 1.25 m, after an initial familiarization period comprising 100 flights per bird made at perch spacing of 12 m. Perch spacing was held at 12 m for the following 2–3 weeks, to allow us to confirm the stability of the behaviour, and was subsequently randomized daily at 5, 7 or 9 m. The juvenile birds flew directly between the perches by flapping for the first few flights of their familiarization period (Fig. 2a) but soon adopted the indirect swooping behaviour characteristic of experienced birds (Fig. 2b–e). Swooping was initiated by jumping forward into a dive involving several powerful wingbeats, which transitioned into an unpowered climb finishing with a rapid pitch-up manoeuvre that ended with the body almost vertical and with the wings outstretched as the feet contacted the perch (Fig. 1). Climbing was mainly executed by gliding, with occasional ventral excursions of the wings that we interpret as corrective control inputs rather than as wingbeats supplying thrust to offset drag (Extended Data Fig. 1).

We summarized the geometry of each trajectory by measuring the position of its lowest point, having low-pass filtered the bird’s vertical position to remove the body oscillations associated with flapping (Methods). We took the trajectory’s lowest point as a proxy for the location of the transition from powered dive to unpowered climb (see Extended Data Fig. 1 for validation) and used a linear mixed effects model to characterize how this location varied with perch spacing over all 1,585 flights, excluding 9 outliers with high residual error. The relative longitudinal position of the observed transition point (marginal mean ± standard error (s.e.) at mean perch spacing, 61.3 ± 1.16% of perch spacing distance) did not vary significantly in relation to perch spacing distance (regression coefficient ± s.e., −0.139 ± 0.15% m^−1^; *t*_(1,574)_ = −0.91; *P* = 0.36). By contrast, the relative depth to which the birds dived (marginal mean ± s.e. at mean perch spacing, 3.22 ± 0.51% of the perch spacing distance) increased linearly with perch spacing distance (regression coefficient ± s.e., 1.06 ± 0.078% m^−1^; *t*_(1,574)_ = 13.5; *P* < 0.0001). In each case, the consistency with which different individuals adopted qualitatively similar swooping behaviour at different perch spacing distances (Fig. 2) suggests that they may have acquired this through individual learning optimizing some common performance objective. What might this objective function be?

## Optimizing the swoop-to-perch manoeuvre

Flying between perches is energetically demanding because of the high aerodynamic power requirements of slow flight, and our hawks were usually panting visibly by the end of a session. Guided by previous work on perching parrotlets^7^, we theorized that the hawks would have learned trajectories minimizing the energetic cost of flying between the perches. An alternative hypothesis is that they learned trajectories minimizing the time of flight^16^, which would make sense for a predator adapted to exploit fleeting feeding opportunities^17^ and would also maximize the net rate of energy gain when flying at speeds below the minimum power speed^16^. Could optimization of either performance objective explain the swooping behaviour that we observed? Diving exploits gravity to reach higher speeds more quickly^29^, so a swooping flight path might be expected to reduce flight duration, analogous to the brachistochrone problem in which a curved path minimizes the time of travel for a particle falling under gravity between two points spaced vertically and horizontally^30^. Diving might also be expected to reduce the energy required for flight, by raising the bird’s airspeed closer to its minimum power speed^29^. It therefore seems intuitive that swooping could reduce both the energetic cost and time of flight.

**Extended Data Fig. 2:**
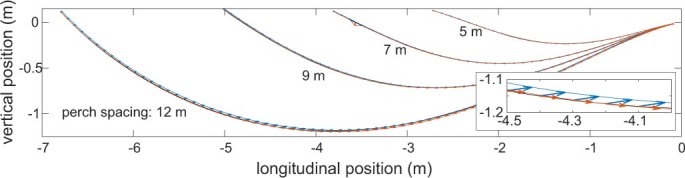
Impact of ground effect on the line of feasible transition points. We assessed the impact of ground effect by re-evaluating the powered dive phase (red) and unpowered glide phase (blue) with an aerodynamic model of ground effect^33^ included for the same set of feasible parameter settings as we had identified previously without ground effect (black). Whereas the powered dive transitions smoothly into the unpowered glide for the original solutions without ground effect (black), the introduction of ground effect without any changes to the model parameters necessarily results in a small mismatch between the two flight phases. The extent of this mismatch is shown by the arrows plotting the change in position of the transition point predicted at the end of the powered dive (red arrows) and the start of the unpowered glide (blue arrows) for trajectories satisfying the take-off and landing constraints. The effect, shown here for the bird “Drogon”, is marginal; the enlarged inset shows the effect on transition points close to the ground at 12 m perch spacing for which the impact of ground effect is greatest.

We used a simplified flight dynamics model to predict how the birds’ performance on these two objectives varied with their behaviour. We used a two-phase model of perching, comprising a powered dive switching to an unpowered glide. For the purposes of the main optimization analysis, we constrained the aerodynamic lift and power to be constant for each flight phase (see Methods for justification and validation). This first-order modelling approach avoids the need to make any detailed assumptions on variation in lift and power as well as the need to model the flapping wing kinematics explicitly. The resulting model captures both the indirect swooping flight behaviour of experienced birds and the direct flight behaviour of naive birds. Our simulations incorporated inter-individual variation in mass, wingspan, wing area, take-off speed and landing speed (Table 1). We modelled aerodynamic drag using a theoretical drag polar parameterized with wind tunnel measurements from Harris’ hawks^31^ and determined thrust as the ratio of power to airspeed. Aerodynamic ground effect is expected to reduce drag when flying over a surface^32,33^, but the birds only dived close to the ground at the perch spacing of 12 m (Fig. 2e) and for so brief a period of time that modelling this^33^ made little difference to the predicted flight trajectory (Extended Data Fig. 2). We therefore ignore ground effect in the optimization, which simplifies its implementation considerably.

**Table 1:** Measurements and model parameters by bird.

Bird	Sex	Age	*m* (kg)	*b* (m)	*S* (m^2^)	${\bar{V}}_{0}$ (m s^−1^)	${\bar{V}}_{{\rm{end}}}$ (m s^−1^)	${\hat{P}}_{{\rm{dive}}}/m$ (W kg^−1^)	*V*_mp_ (m s^−1^)
Drogon	Male	Juv.	0.660	1.01	0.1895	3.9	2.3	23.2	10.2
Rhaegal	Male	Juv.	0.620	1.02	0.1918	4.0	2.5	18.9	10.8
Ruby	Female	Adult	0.874	1.08	0.2146	3.9	2.3	22.4	9.8
Toothless	Male	Juv.	0.738	1.07	0.2098	3.8	2.5	22.7	10.1

Morphological data and summary flight statistics for each bird. Juv., juvenile; *m*, total mass; *b*, wingspan; *S*, wing area; ${\bar{V}}_{0}$, mean observed take-off speed; ${\bar{V}}_{{\rm{end}}}$, mean observed landing speed; ${\hat{P}}_{{\rm{dive}}}/m$, best-fitting specific power setting; *V*_mp_, estimated minimum power speed in level flight.

**Extended Data Fig. 3:**
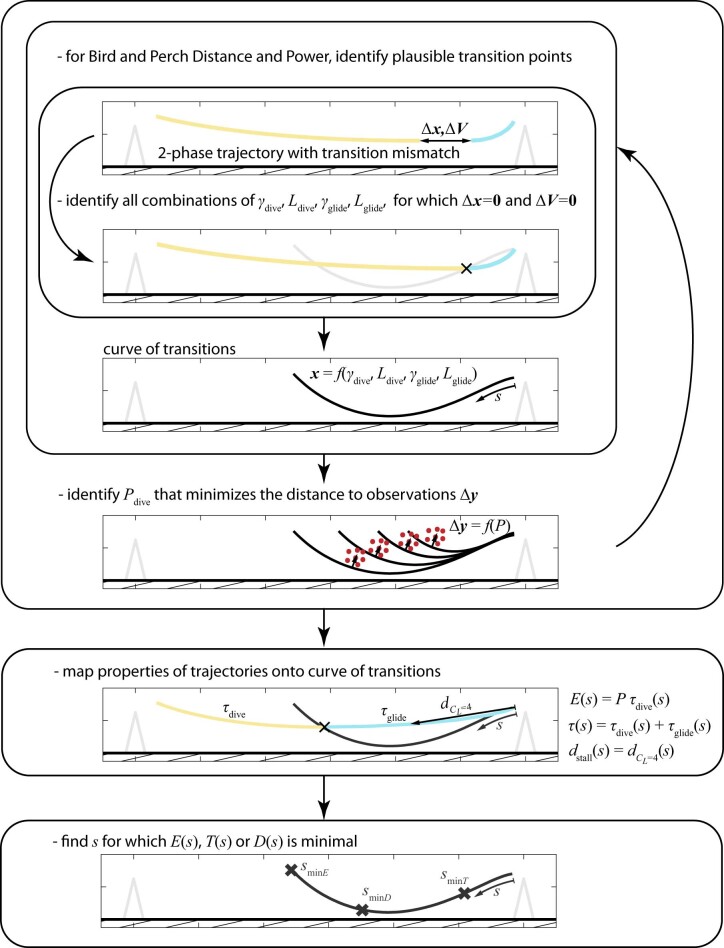
Flow diagram summarising the key steps in model optimization. We begin with a parameter search to find combinations of parameter values {*γ*_end_,*L*_glide_} and {*γ*_0_,*L*_dive_,} for which the end of the powered phase matches the start of the unpowered phase at a given power setting (*P*_dive_). This search defines the set of feasible transition points for that power setting, and is done separately for each combination of perch spacing and bird identity. We then calibrate the power setting for each bird separately over all perch distances, such that the distance between the observed transition points and the respective line of feasible transition points is minimized. Finally, we map the relevant properties of these feasible trajectories onto the line of feasible transition points as a function of the position along this line (*s*), and identify the optimal location of the transition point that minimizes each of the performance objectives (i.e. energy expenditure *E*, time of flight *τ*, and stall distance *d*_stall_).

For a given take-off speed (*V*_0_), the model’s powered phase is parameterized by its initial dive angle (*γ*_0_), constant lift setting (*L*_dive_) and constant power setting (*P*_dive_). The entry conditions for the glide phase are given by the transition speed (*V*_T_) and the position (*x*_T_,*y*_T_) of the bird at the end of this powered dive phase, such that the constant lift setting for the unpowered glide phase (*L*_glide_) is uniquely determined by the constraint that the bird must intercept the perch at some given landing speed (*V*_end_). Enforcing this constraint with respect to the bird’s mean landing speed (Table 1) allowed us to identify a set of feasible parameter settings {*γ*_0_,*L*_dive_,*P*_dive_} for each bird and each perch spacing distance that would bring the bird safely to the perch (Extended Data Fig. 3). For any given power setting *P*_dive_, these feasible parameter settings map onto a line of feasible transition points {*x*_T_,*y*_T_} characterizing the full range of feasible perching trajectories at that power output (Extended Data Fig. 3). We determined the best-fitting power setting separately for each bird by minimizing the mean squared distance between the observed transition points and the line of feasible transition points (Extended Data Fig. 3). This yielded specific power estimates ranging from 18.9 to 23.2 W kg^−1^ (Table 1) for the powered dive, which is comfortably below the maximum power that Harris’ hawks have available to use when climbing^34,35^.

## Swooping does not minimize time or energy

**Fig. 3:**
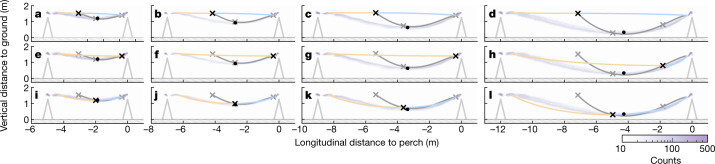
Optimal perching trajectories minimizing different cost functions at different perch spacings. **a**–**l**, Thick coloured lines show trajectories predicted for the juvenile bird ‘Drogon’ at perch spacing of 5 m, 7 m, 9 m or 12 m under the two-phase perching model, comprising a powered dive (yellow line) transitioning into an unpowered glide (cyan line), and minimizing energetic cost (**a**–**d**), flight duration (**e**–**h**) or distance flown after the stall (**i**–**l**). The location of the optimal transition point (black cross) along the line of feasible transition points (grey line) is only close to the mean observed transition point (black dot) if the stall distance is optimized (**i**–**l**); observed trajectories are shown as a spatial histogram (lilac shading).

The line of feasible transition points ranges from almost-level flight trajectories involving a short powered phase and a long glide phase (Fig. 3a–d) through to almost-level flight trajectories involving a long powered phase and a short glide phase (Fig. 3e–h); intermediate transition points are associated with deep swooping trajectories resembling those observed in experienced birds (Fig. 3i–l). For the same dive power *P*_dive_, alternative transition points above the line require reverse thrust to be added on the glide phase to arrive at the same safe landing speed, whereas points below the line require additional thrust to reach the perch. Such alternatives are outside the scope of the model, owing to their requirement for positive or negative power on the glide, but may be feasible for birds capable of touching down in hover. The optimization yields some unexpected findings. First, although diving more steeply allows faster speeds to be reached sooner in the powered phase as expected, shortening the glide phase proves more effective in reducing flight duration. The time-optimal solution therefore involves a long, shallow powered dive and a short glide (Fig. 3e–h). Second, although the efficiency of lift production is enhanced at faster speeds as expected, more energy is needed in a deeper dive because of the higher lift required to swoop up to the perch. The energy-optimal solution therefore involves an almost-level flight trajectory, with a short, flat powered phase and a long glide (Fig. 3a–d). It follows that neither time nor energy minimization straightforwardly explains the deep swooping flight behaviour that our birds acquired, at least not under the modelled constraints of constant lift and power.

**Fig. 4:**
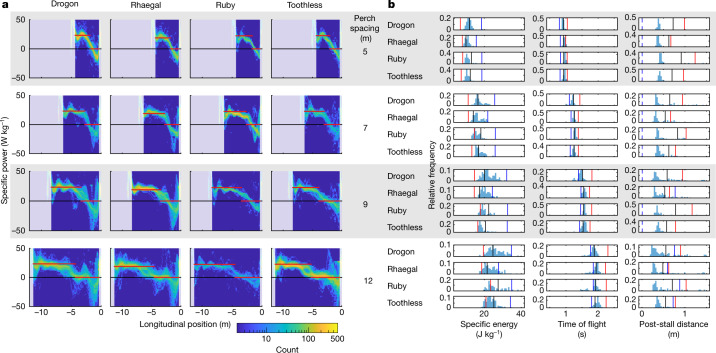
Comparison of observed and modelled flight performance. **a**, Two-dimensional histograms of estimated power (*P*) over all observed flight trajectories, estimated under the drag model given the observed variation in flight velocity. Red lines plot the fixed power settings assumed in the main optimization analysis, with *P* = *P*_dive_ in the powered dive phase and *P* = 0 in the unpowered glide phase. **b**, Histograms showing the birds’ observed or estimated performance against each of the three objectives of energetic cost, time of flight and distance flown after the stall over the trajectories observed at each combination of bird identity and perch spacing. Vertical lines indicate the corresponding model optima subject to the constraints of constant lift and power (red, energy optimal; blue, time optimal; black, stall optimal). Energetic cost was evaluated by integrating the positive power required to offset the modelled drag between the defined take-off and landing points of the observed flight trajectories and is expressed as specific energy relative to body mass; time of flight was assessed as the time difference between the defined take-off and landing points; and stall distance was defined as the greatest distance from the landing point for which *C*_*L*_ > 4 in the glide phase (dashed lines indicate transitions where the glide phase initiated at *C*_*L*_ > 4).

To verify that this result was not merely an artefact of model reduction, we compared the time and energy minima predicted assuming constant lift and power to the observed and estimated distributions of time and energy cost across flights (Fig. 4). To do so, we assessed the duration of the observed flights directly and evaluated their energetic cost by using the drag polar to estimate the time history of the aerodynamic power output from the time history of the observed flight velocity. Apart from confirming the validity of our constant power approximation (Fig. 4a), this analysis showed that the time and energy that the birds expended when perching were suboptimal when compared with their respective minima under the constraints of constant lift and power (Fig. 4b). Hence, although it is plausible that trajectories of shorter duration or lower energetic cost might exist given variable lift and power, these are not the trajectories that the birds used. We conclude that the birds did not shape their flight behaviour to minimize either time or energy alone. It is possible in principle that the birds jointly optimized time and energy, but, in the absence of any prior expectation of what form this trade-off might take, it is reasonable to ask whether the birds were optimizing a different kind of performance objective altogether. What might this be?

## Swooping minimizes stall distance

**Extended Data Fig. 4:**
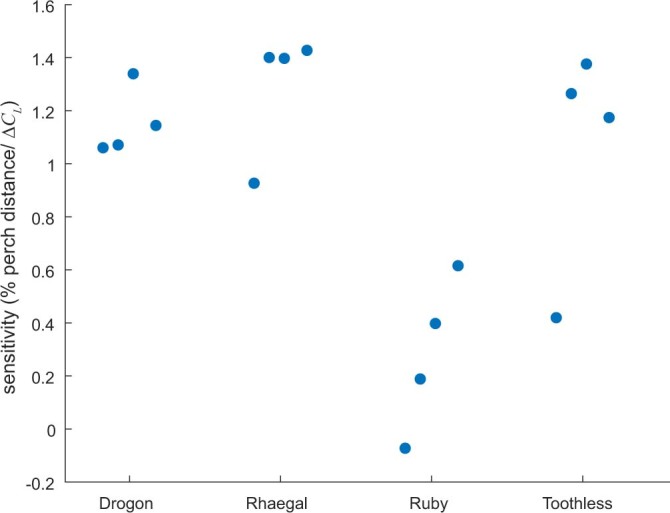
Effect of lift coefficient threshold on location of transition points minimizing distance flown post-stall. In the main text, the location of the stall-optimal transition point is determined by minimizing the distance flown at lift coefficients *C*_*L*_ > 4. Decreasing this penalty threshold causes the optimum to move closer to the landing perch along the line of feasible transition points, eventually reaching a point of minimum distance from the landing perch at some specific decrement ∆*C*_*L*_, depending on individual bird and perch spacing. We quantified the sensitivity of the solution to the choice of penalty threshold by expressing the maximal displacement of the optimal transition point along the line of feasible transitions points relative to the corresponding decrement in penalty threshold ∆*C*_*L*_. The points for each bird represent 5 m, 7 m, 9 m, and 12 m perch spacing, and the displacement of the optimal transition point is normalized by perch spacing distance. Note the robustness of the location of the stall-optimal transition point to the choice of penalty threshold (displacement sensitivity < 1.5% of perch spacing distance per unit decrement in the penalty threshold value of *C*_*L*_).

Minimizing either time or energy under the model requires high lift coefficients *C*_*L*_ = 2*L*/(*ρV*^2^*S*) to be sustained in the glide phase, where *L* is lift, *ρ* is air density, *V* is airspeed and *S* is wing area. This is necessary to induce the high drag needed to brake with high force in the short glide phase minimizing flight time (Fig. 3e–h) and to support body weight at the low airspeeds sustained in the long glide phase minimizing energetic cost (Fig. 3a–d). High lift coefficients may be achieved transiently during unsteady perching manoeuvres^36,37^, but stall cannot be delayed indefinitely and will compromise control authority when it occurs^10,12,20,24^. We therefore propose that birds aim to minimize the distance from the perch at which high lift coefficients become necessary to complete the glide phase. We tested this hypothesis by identifying the transition point that minimized the distance flown at *C*_*L*_ > 4. We set this threshold lift coefficient high to avoid penalizing the comparably high lift coefficients achieved transiently in a rapid pitch-up manoeuvre^38^, but found the resulting solutions to be robust to the selection of lower threshold lift coefficients (Extended Data Fig. 4). Exceeding the threshold lift coefficient on the glide need not mean that a trajectory will fail, but rather that flapping may become necessary to maintain control authority after the stall, at a cost that will presumably scale with the distance remaining to the perch. This cost could manifest itself in several ways, including through (1) the control effort needed to steer a trajectory under high aerodynamic load; (2) the energy needed to flap the wings to achieve such high loads; and (3) the requirement to stabilize gaze against wingbeat oscillations on final approach.

**Fig. 5:**
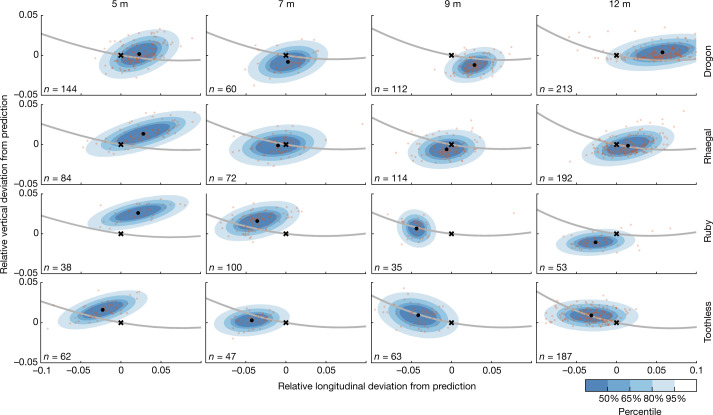
Fit of observed transition points to optima minimizing distance flown after the stall. At each combination of bird and perch spacing, the *n* observed transition points (opaque dots) are compared with the optimal transition point predicted to minimize stall distance under the model (black cross). The black dot denotes the sample mean for each test condition, coloured contours denote the 50th to 95th percentiles of a bivariate normal distribution fitted using the sample mean and covariance matrix within each group at 15% intervals and the grey line denotes the line of feasible transition points predicted under the model. Distance deviation is shown as a proportion of perch spacing distance.

**Extended Data Fig. 5:**
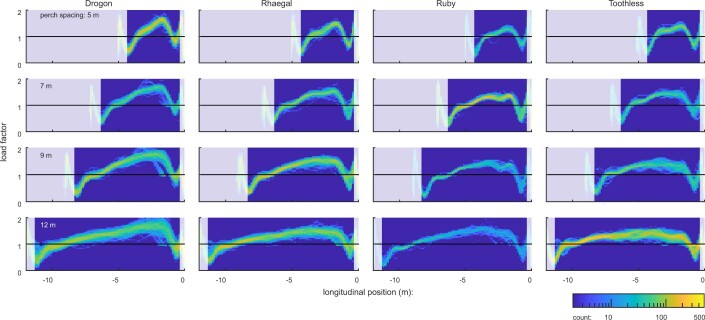
Estimated variation in lift over the observed flight trajectories. Two-dimensional histogram of the variation in the load factor $L/(mg)$ estimated for the observed flight trajectories given their observed variation in flight velocity. Note how the lift ramps up quickly after take-off, and drops sharply before landing; between these points, the lift increases gradually.

**Extended Data Fig. 6:**
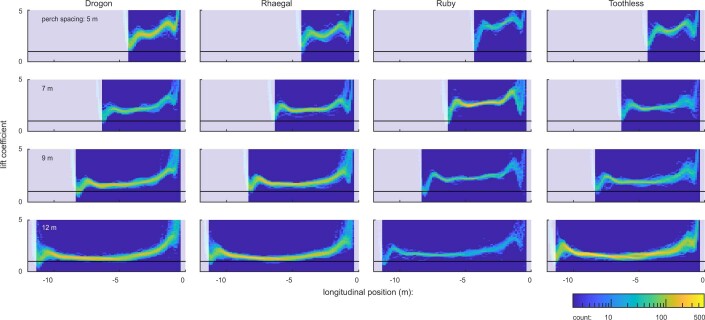
Estimated variation in lift coefficient over the observed flight trajectories. Two-dimensional histogram of the variation in the lift coefficient *C*_*L*_ estimated for the observed flight trajectories given their observed variation in flight velocity. Note how the lift coefficient increases quickly from around zero at take-off to reach a local maximum close to the starting perch. A second local maximum (3 < *C*_*L*_ < 5) occurs shortly before contact with the landing perch. The further increases in estimated lift coefficient seen at the end of the recording window are presumably an artefact of contact forces.

Minimizing the distance flown at *C*_*L*_ > 4 produced deep swooping trajectories (Fig. 3i–l) resembling those observed in experienced birds (Fig. 2b–d), with transition points whose predicted locations were statistically indistinguishable from those that we observed (linear mixed effects model of deviation from predictions; mean longitudinal deviation, −0.52% (95% confidence interval (CI), −2.0%, 0.97%); mean vertical deviation, 0.41% (95% CI, −0.010%, 0.91%)). The close quantitative match of observations and predictions across all combinations of perch spacing and bird identity (Fig. 5) is therefore consistent with our hypothesis that the hawks learned perching trajectories minimizing stall distance. To verify the robustness of this conclusion, we relaxed the model’s constraints of constant lift and power by evaluating the distance that the birds would have flown at *C*_*L*_ > 4 given the time history of their observed flight velocity. In most cases, the birds substantially outperformed the model (Fig. 4b), which is just as we would expect if they had optimized any actual variation in their lift (Extended Data Fig. 5) to maintain lower lift coefficients (Extended Data Fig. 6) and to minimize the distance flown after the stall.

## Conclusions

In summary, our birds learned swooping trajectories that enabled them to reach the perch in a glide, by postponing stall until they were as close to the perch as possible. This heuristic is expected to be of particular importance to larger birds whose power margin prohibits hovering^9^, but may also be relevant to smaller birds that choose to swoop rather than to hover when perching. Although this behaviour does not necessarily minimize energy consumption (Fig. 4b), it avoids any hazardous loss of control authority and may aid visual fixation of the perch by avoiding wingbeat perturbations on approach (Extended Data Fig. 1). How might this heuristic principle be implemented in practice? Learning to minimize stall distance on the fly requires aeromechanical information (for example, from feather^1,2^ or muscle^39^ proprioceptors) and distance information (for example, from static visual^5^ or optic flow^7,11,40^ cues) to be combined. Fly-by-feel concepts^41–45^ may therefore prove critical to the learning and control of perching in autonomous vehicles. Moreover, it seems likely that our birds would have learned the optimized position of the transition point as a virtual target for trajectory control, analogous to the ‘entry gate’ approach adopted in one recent implementation of autonomous perching^18^. Our findings suggest a heuristic for guiding reinforcement learning of autonomous perching, for which the identification of an appropriate reward function is critical^18^.

## Methods

### Experimental set-up

We flew *n* = 4 captive-bred Harris' hawks (*Parabuteo unicinctus*) between two 1.25-m-high A-frame perches positioned 5, 7, 9 or 12 m apart in a purpose-built motion capture studio (Fig. 1 and Supplementary Video 1). The sample comprised an experienced adult female (age, 7 years) and three inexperienced juvenile males (ages, ≤0.5 years) (Table 1). The sample size was determined in line with related work on perching^7^ and on grounds of practicality (that is, the sample size was not predetermined using statistical methods), noting that each hawk required housing separately in its own large aviary and could only be flown until satiation. The inexperienced birds had only previously flown during a brief period of fitness training conducted immediately before the experimental trials. The flights were undertaken in a windowless hall measuring 20.2 m × 6.1 m, with a minimum ceiling height of 3.8 m and walls hung with camouflage netting to provide visual contrast. Flicker-free LED lights provided a mixture of direct 4,000 K lighting and indirect 5,000 K lighting at approximately 1,000 lux, designed to mimic overcast morning or evening light.

### Experimental design

We collected trajectory data over an experimental period comprising 5–6 weeks of flight testing per bird. Each bird was flown individually between the perches, on a variable number of flights of up to approximately 50 per session. The birds were motivated to fly from the take-off perch by the presentation of a small food reward on the landing perch and usually responded immediately to this stimulus. The session was ended if the bird appeared tired or lacking in motivation, and the birds received a larger food reward at the end of the session. The birds were initially flown with perch spacing of 8 m to introduce them to the testing environment and establish the measurement protocol. Perch spacing was then held at 12 m for approximately 2–3 weeks to allow us to identify when the birds’ behaviour had stabilized, before being randomized at 5, 7 or 9 m daily thereafter. All individuals were flown repeatedly under all experimental conditions. Blinding the experimenters to the test condition was not possible because the experiment involved flying each bird between two perches at a fixed spacing distance that was varied experimentally. The identity of the bird and the spacing of the perches were therefore known to the experimenters and are implicit in the resulting data structure, but the physical nature of the measurements means that this is unlikely to have biased the results. We treated the first 100 flights at a perch spacing of 12 m for each bird as an initial familiarization period, by the end of which their flight behaviour had stabilized. The flights from this familiarization period are not included in the main analysis but are illustrated in Fig. 2a for the bird Toothless.

### Ethics statement

This work was approved by the Animal Welfare and Ethical Review Board of the Department of Zoology, University of Oxford, in accordance with university policy on the use of protected animals for scientific research, permit no. APA/1/5/ZOO/NASPA, and was considered not to pose any significant risk of causing pain, suffering, damage or lasting harm to the animals.

### Motion capture

We reconstructed the birds’ flight trajectories using a 20-camera motion capture system (Vicon Vantage 16, Vicon Motion Systems), mounted 3 m above the floor on scaffolding fixed around the walls. The motion capture system was turned on at least 1 hour before the start of the experiments and was calibrated shortly before the first session (Vicon Active Calibration Wand), using Vicon Nexus 2 software for data acquisition. The motion capture cameras were set to record at 120 or 200 Hz under stroboscopic 850-nm infrared illumination, well outside the visible spectrum of these birds^46^, and a set of four high-definition video cameras (Vicon Vue) recorded synchronized reference video at 120 or 100 Hz, respectively. Each hawk was fitted with a rigid marker template comprising four 6.4-mm-diameter spherical retroreflective markers (Fig. 1) worn on a falconry backpack secured by a pair of Teflon ribbons (TrackPack Mounting System, Marshall Radio Telemetry). The birds sometimes wore other retroreflective markers carried on fittings on the head, wings or tail (Supplementary Video 1), but these were not included in the present analysis. A pair of 9.5-mm-diameter spherical retroreflective markers was fixed to either end of each perch to identify the perch axis.

### Marker reconstruction

We used Vicon Nexus 2.7.6 software to reconstruct the positions of the markers within the flight volume, using a coordinate system corresponding to the principal axes of the flight hall. We removed any flights for which there were long sections of missing data or for which the bird did not land at the perch, resulting in a sample of *n* = 1,585 complete flight trajectories suitable for analysis. This comprised *n* = 649 flights recorded at perch spacing of 12 m, *n* = 324 flights at perch spacing of 9 m, *n* = 279 flights at perch spacing of 7 m and *n* = 333 flights at perch spacing of 5 m. The backpack and tail mount markers were usually visible on >70% of the recorded frames, but, because of a challenging combination of dense marker placement, intermittent marker occlusion and high-speed motion, the proprietary marker tracking algorithms were not uniformly successful in matching markers between frames. In addition, patches of specular reflection sometimes appeared as spurious markers. Consequently, although the Nexus software reconstructed the positions of all visible markers to a high degree of accuracy, it was not always able to label each marker reliably or to identify every marker on every frame. We therefore wrote a custom script in MATLAB v2018a (Mathworks) that analysed the pattern of pairwise distances between markers in the rigid templates to label the anonymous markers.

### Marker labelling

The anonymous markers were labelled separately for each frame by using Procrustes analysis to match any visible markers to the known backpack template. We used the centroid of the resulting set of candidate backpack markers as an initial estimate of backpack position and fitted a quintic spline to interpolate its position on frames with missing data. We then used our initial or interpolated estimate of the backpack’s position on each frame to define a search volume matched to the size of the backpack template and labelled any other markers falling within this search volume as candidate backpack markers. This two-stage labelling approach was able to accommodate missing markers and occasional spurious markers and successfully identified the correct number of markers in >80% of all frames in which the backpack markers were visible. As the backpack sat directly between the scapulars, we took the centroid of the candidate backpack markers to approximate the position of the bird’s centre of mass and estimated its velocity and acceleration by fitting and differentiating the smoothest quintic spline function passing through the positions measured on each frame.

### Trajectory analysis

The birds’ characteristic swooping behaviour involves a powered dive transitioning into an unpowered climb (Fig. 1). Because the birds morphed smoothly from flapping to gliding, it was not possible to identify a unique point at which this transition occurred with reference to the wing kinematics. Instead, we identified the transition as occurring at the lowest point in the bird’s flight trajectory (Supplementary Video 1), which we determined having low-pass filtered the trajectory to remove body oscillations due to flapping (forwards–backwards filtering implemented using a sixth-order Butterworth filter with a 2-Hz cut-off; supporting code). Identifying the transition from powered to unpowered flight as the lowest point of the trajectory makes sense from first principles (see ‘Flight dynamics model’ below) and is supported empirically by visual inspection of the heave oscillations removed by filtering the motion capture data (Extended Data Fig. 1), which confirms that these oscillations mainly occur before the transition point.

### Take-off and landing

Each flight was initiated by a take-off jump during which the feet remained in contact with the perch. This jump ended with a jerk as the feet released from the perch, but the noise associated with the measured acceleration, particularly during flapping, makes it an unreliable marker of the onset of the flight phase. We therefore defined flight proper as beginning when the backpack reached a horizontal distance of 0.65 m from the take-off perch axis, this threshold distance being determined through visual inspection of the angular acceleration traces over many flights. The point of contact with the landing perch was likewise associated with a pronounced linear and angular acceleration, but for similar reasons we define the flight proper as ending when the backpack reached a horizontal distance of 0.35 m from the landing perch axis. The difference in these two threshold distances relates to the fact that the bird’s legs are extended caudad at take-off and ventrad at landing. We found that the backpack was located approximately 0.30 m above the perch at take-off and approximately 0.15 m above the perch at landing, which we used to define the initial and terminal conditions for the flight dynamics modelling.

### Flight dynamics model

We built a simplified flight dynamics model to assess what performance objectives were optimized by the birds’ swooping flight trajectories (supporting code). From first principles, any flight behaviour that begins and ends in a stationary state must minimally involve an acceleration phase followed by a deceleration phase, and, as the perch spacing was insufficient for the birds to reach their minimum power speed, it is safe to assume that there will have been no intermediate steady flight phase. We therefore model perching as a two-phase flight behaviour. Gravity will assist both phases in their entirety if the bird dives throughout the acceleration phase and climbs throughout the deceleration phase. For continuity, we therefore assume that the transition between the phases occurs in horizontal flight.

A centripetal lift force (*L*) is necessary to produce the convex trajectory that these assumptions imply, and a tangential drag force (*D*) is assumed to dissipate kinetic energy throughout the flight. The integrated drag losses must be exactly balanced by the bird’s integrated aerodynamic power output (*P*), net of any transfer of gravitational potential energy. As a first-order modelling approach, we assume constant power output (*P* = *P*_dive_) on the acceleration phase and zero power output (*P* = 0) on the deceleration phase, which we thereby treat as a glide. Likewise, we assume that the lift force remains constant at a setting (*L* = *L*_dive_) on the acceleration phase and *L* = *L*_glide_ on the deceleration phase. In other words, we assume that lift and power remain constant at their mean values for each phase. This represents the simplest possible implementation of the physical constraints on the problem, but we relax these assumptions later to assess the validity of the model (see below).

The bird’s aerodynamic power output implies a tangential aerodynamic thrust force *T* = *P*⁄*V*, where *V* is the bird’s airspeed neglecting any induced velocity component. We model the opposing aerodynamic drag as

1 $$D=kL{\left(-\frac{1}{2}+\sqrt{\frac{1}{4}+{\left(\frac{2L}{\rho {V}^{2}\pi {b}^{2}}\right)}^{2}}\right)}^{\frac{1}{2}}+\frac{1}{2}\rho {V}^{2}(S{{C}_{D}}_{{\rm{p}}{\rm{r}}{\rm{o}}}+{S}_{{\rm{b}}}{{C}_{D}}_{{\rm{p}}{\rm{a}}{\rm{r}}})$$

where *ρ* = 1.23 kg m^−3^ is air density and where *S*_b_ = 0.00813*m*^2/3^ is an empirical scaling relationship^47^ modelling body frontal area *S*_b_ as a function of body mass *m*. Here *b* is wingspan and *S* is wing area, both of which are assumed to be maximal throughout the manoeuvre (Table 1). The first term of equation (1) represents the induced drag and models how lift production is powered by the kinetic energy of the flow past the wings in a form that ensures that this energy cost remains bounded at low speeds^48^. The second term of equation (1) represents the contributions of profile and parasite drag^31^. We modelled the dimensionless induced drag factor *k* = 1.623 and the profile drag coefficient ${{C}_{D}}_{{\rm{p}}{\rm{r}}{\rm{o}}}=0.003$ as empirical constants. These were fitted by regressing the drag measurements of an empirical glide polar for a Harris’ hawk^31^ against the predictors on the right-hand side of equation (1) while holding the parasite drag coefficient fixed at an estimated value^49^ of ${{C}_{D}}_{{\rm{p}}{\rm{a}}{\rm{r}}}=0.2$. Treating ${{C}_{D}}_{{\rm{p}}{\rm{r}}{\rm{o}}}$ and ${{C}_{D}}_{{\rm{p}}{\rm{a}}{\rm{r}}}$ as constants means that equation (1) does not capture the high profile and parasite drag produced at high angles of attack, but the expression for the induced drag predicts high drag at high lift coefficients *C*_*L*_ = 2*L*/(*ρV*^2^*S*) and can therefore be treated as capturing this effect by proxy.

With these assumptions, the rate of change in airspeed and flight path elevation angle (*γ*) can be written using Newton’s second law as

$$\dot{V}=\frac{1}{m}\left(\frac{P}{V}-D\left(L,V\right)\right)-g{\rm{\sin }}\gamma $$

2a,b $$\dot{\gamma }=\frac{1}{V}\left(\frac{L}{m}-g{\rm{\cos }}\gamma \right)$$

where *g* is gravitational acceleration and *m* is the bird’s mass. We modelled the resulting flight trajectories in lab-fixed Cartesian coordinates (*x*,*y*) by coupling equations (1) and (2) for $\dot{V}$ and $\dot{\gamma }$ with the component kinematics equations:

$$\dot{x}={V}_{x}$$

$$\dot{y}={V}_{y}$$

$${\dot{V}}_{x}=\frac{\dot{V}}{V}{V}_{x}-\dot{\gamma }{V}_{y}$$

3a&x02013;d $${\dot{V}}_{y}=\frac{\dot{V}}{V}{V}_{y}+\dot{\gamma }{V}_{x}$$

with $V=\sqrt{{V}_{x}^{2}+{V}_{y}^{2}}$. We integrated these ordinary differential equations numerically using the ode45 solver in MATLAB, which is based on an explicit Runge–Kutta (4,5) formula, the Dormand–Prince pair.

### Trajectory simulations

We simulated each bird separately to account for inter-individual variation in flight morphology (Table 1). We matched the initial speed *V*(0) of the simulations to the mean take-off speed ${\bar{V}}_{0}$ observed for each bird at the threshold horizontal distance of 0.65 m from the take-off perch (Table 1). We treated the initial dive angle *γ*(0) = *γ*_0_ as a free parameter *γ*_0_ < 0, so the initial conditions for integrating equation (3) were ${V}_{x}={\bar{V}}_{0}\,\cos {\gamma }_{0}$ and ${V}_{y}={\bar{V}}_{0}\,\sin \,{\gamma }_{0}$ with *x*(0) = 0.65 m, *y*(0) = 1.55 m. Coupling equations (1) and (2) with equation (3), any given combination of parameter settings {*γ*_0_,*L*_dive_,*P*_dive_} defines a unique powered dive trajectory. A subset of these powered dive trajectories pass through the horizontal, in the sense of having a point (*x*_T_,*y*_T_) where *V*_*y*_ = 0 with *V*_T_ = *V*_*x*_ > 0, which is satisfied when *L*_dive_ > *mg* cos *γ*_0_ (equation (2b)). This represents the set of reachable combinations of position and speed at which the transition from powered dive to unpowered glide can occur in horizontal flight under the model.

The initial conditions for the glide phase are given by the position (*x*_T_,*y*_T_) and velocity (*V*_T_,0) of the bird at this transition point. The corresponding parameter settings {*γ*_0_,*L*_dive_,*P*_dive_} for the powered dive phase therefore define a family of possible flight trajectories for the glide phase, which is in turn parameterized by its own constant lift setting (*L* = *L*_glide_). Hence, for any given combination of parameter settings {*γ*_0_,*L*_dive_,*P*_dive_}, we are left to solve for the unique value of *L*_glide_ that produces a trajectory intercepting the point of contact with the landing perch at *x* = *s* − 0.35 m and *y* = 1.40 m, where *s* is the perch spacing. In practice, there are only certain combinations of parameter settings {*γ*_0_,*L*_dive_,*P*_dive_} that will bring the simulated bird to the landing perch at a realistic speed. It therefore proved most efficient to solve the glide phase backwards in time from the point of contact with the landing perch and to match the solutions for the two flight phases at the transition point (supporting code). We fixed the initial speed of this backwards simulation of the glide phase to the mean landing speed ${\bar{V}}_{{\rm{end}}}$ observed for each at the threshold horizontal distance of 0.35 m from the landing perch (Table 1). For the purposes of finding matching solutions, we treated both the flight path angle at the point of contact (*γ*_end_) and the constant lift setting for the glide phase (*L*_glide_) as free parameters. These parameters {*γ*_end_,*L*_glide_} then become fixed for a given combination of parameter settings {*γ*_0_,*L*_dive_,*P*_dive_} once the matching solution for the powered dive phase is found.

### Feasible trajectory search

We define feasible trajectories under the model as those that bring an individual bird to its landing perch at the same mean speed and position as we observed in the experiments. For any given constant power setting *P*_dive_, this constraint defines a line of feasible transition points corresponding to a line of feasible parameter settings {*γ*_0_,*L*_dive_}. We implemented the search for feasible transition points as a constrained minimization problem solved using an interior-point algorithm in MATLAB 2020a. For a given constant power setting *P*_dive_, we constrained the difference in transition point position (*x*_T_,*y*_T_) and velocity (*V*_T_,0) between the end of the powered dive phase and start of the unpowered glide phase to be zero and solved for the parameter settings {*γ*_0_,*L*_dive_} and {*γ*_end_,*L*_glide_} that would have placed the transition point at the landing perch. We then took these parameter settings as initial values when solving for the parameter settings that would have caused the transition point to be placed a small increment ahead of the perch, which we set as the target of the minimization. We repeated this process to place the transition point another small increment in distance ahead of the perch, inheriting the parameter settings of the previous solution as initial values for the next round until the complete line of feasible transition points had been found (Extended Data Fig. 3). Other transition points falling close to this line could also be physically feasible in the sense of bringing the bird to the landing perch, but these will be associated with higher or lower speeds than those observed at the point of contact. Finally, we determined the constant power setting *P*_dive_ for each bird by finding the value that minimized the mean squared distance between the observed transition points and the line of feasible transition points (Extended Data Fig. 3).

### Trajectory optimization

The unique mapping that exists between the parameter settings {*γ*_0_,*L*_dive_,*P*_dive_} and the transition point {*x*_T_,*y*_T_,*V*_T_} for each bird means that any property of a given flight trajectory is also a property of its transition point. This includes the duration (*τ*) and energetic cost (*E*) of the flight and the distance flown after the stall (*d*_stall_), each of which may be considered a candidate objective function for minimization. We identified the optimal transition point at which each of these objectives was minimized by a direct search along the line of feasible transition points. Under the two-phase model of perching, the duration of a flight trajectory is implicit in its solution as *τ* = *τ*_dive_ + *τ*_glide_. Minimizing the total flight duration *τ* therefore entails jointly minimizing the duration of the powered dive phase *τ*_dive_ and unpowered glide phase *τ*_glide_. By contrast, given the constant power assumption, the energetic cost of a flight trajectory is simply *E* = *P*_dive_*τ*_dive_, so, for a given constant power setting *P*_dive_, minimizing the energetic cost of the flight is equivalent to minimizing the duration of the powered dive phase *τ*_dive_ alone. Wing stall is a complex phenomenon, so we did not model its effects directly. However, because lift varies as *L* = *ρV*^2^*SC*_*L*_/2, stall is implicit in the very high values of the lift coefficient *C*_*L*_ that are needed to meet the constant lift requirement (*L* = *L*_glide_) at the low speeds *V* reached as the bird decelerates on approach to the perch. Minimizing the distance flown after the stall therefore amounts to penalizing flight at values of *C*_*L*_ exceeding some specified threshold, which we implemented by minimizing the distance flown at *C*_*L*_ > 4. In practice, the predicted location of the optimal transition point was robust to this choice of threshold, moving ≤1.5% of perch spacing distance per unit decrement in the threshold value of *C*_*L*_ (Extended Data Fig. 4).

### Model validation

Our modelling makes considerable simplifications with respect to the real-world flight dynamics and control inputs, by treating the bird as a point mass and constraining lift, thrust and drag production in particular ways (see ‘Flight dynamics model’ above). We make these simplifications because we aim for a generalized parameterization that appropriately captures fundamental properties of the observed flights while allowing us to assess performance against the different objective functions even outside of the observed range of flight behaviour. In so doing, we ignore the complications of flapping flight dynamics, which would result in non-monotonic accelerations and reduced propulsive and aerodynamic efficiency. Nevertheless, by low-pass filtering the motion capture data, we already observe flight performance smoothed across wingbeats, and, by adjusting the model’s constant power setting to best match our observations, we implicitly calibrate the unknown reductions in propulsive and aerodynamic efficiency.

The model bird has limited control options compared with a real bird, with lift and power assumed to be held constant on each flight phase in the main optimization analysis. We tested the validity of these assumptions by estimating the actual variation in lift and power for the observed flight trajectories. We used central differencing of the filtered trajectories to estimate the time history of velocity and acceleration for each flight. After subtracting gravity from the estimated acceleration, we decomposed the acceleration into its centripetal and tangential aerodynamic components corresponding to lift and net thrust-drag, respectively. We then used equation (1) to estimate the drag for each time instant and solved for the power from the observed tangential acceleration (equation (2a)). We found that the estimated power output remained approximately constant during the powered dive phase, closely matching the power settings that we had fitted to the observed transition points for each bird (Fig. 4a). The negative power estimate at the end of the glide phase suggests a deficiency in the model’s drag prediction at high angles of attack, but the near-zero power predicted over most of the glide phase on the longer flights confirms that the model performs as intended at lower angles of attack. For the purposes of evaluating the total energetic cost of each flight, we integrated only the positive power contributions between the defined points of contact.

We found that the lift did not remain constant as our simplified model assumes, ramping up quickly after take-off and dropping sharply before landing; between these points, the lift increased more gradually (Extended Data Fig. 5). Because of this variation in lift production, the estimated lift coefficients remain below the default threshold lift coefficient for most of the flight, with *C*_L_ < 4 for much of the glide phase (Extended Data Fig. 6). Notwithstanding this actual variation in lift within a flight, both the overall depth of the trajectory and the total induced drag cost depend predominantly and unambiguously on the mean lift. By varying the lift within each flight phase, a bird could only subtly outperform our simplified model, whereas implementing this variation in the optimization would increase the dimension of the parameter space and would require the introduction of more speculative assumptions.

### Statistical analysis

We modelled the longitudinal and vertical position of the observed transition points as a proportion of perch spacing distance using a linear mixed effects model (fit1me) in MATLAB 2020a. We treated the mean centred perch distance as a covariate and individual as a random effect, such that RelativePosition ~ 1 + MeanCentredPerchDistance + (1 + MeanCentredPerchDistance|BirdID), using two-tailed *P* values for statistical inference. The model for relative longitudinal position identified 9 of the 1,585 observed transition points as outliers, with residuals more than three times the residual standard deviation. Two of these outliers were attributable to motion capture error; the remainder corresponded to non-swooping flight behaviours (7 flights) and/or trajectories at perch spacing of 5 m for which the transition occurred at a local rather than global minimum in height (3 flights). As these outliers are unrepresentative of the swooping behaviour analysed in this paper, we excluded them from this and subsequent analyses, leaving a slightly smaller sample of 1,576 flights (supporting code).

To test how well the flight dynamics model predicted the observed transition points, we fitted the longitudinal and vertical distances between the predicted optima and the observed transition points using a linear mixed effects model. We treated the combination of perch distance and individual as a random effect to account for the single prediction for each experimental condition, such that Distance ~ 1 + (1|PerchDistance:BirdID). In this model, a significant intercept indicates a systematic deviation between the observed and predicted transition points. We computed the sample mean and covariance matrix of the longitudinal and vertical position of the observed transition points at every combination of individual identity and perch distance and generated the percentiles of the corresponding bivariate normal distribution by computing their squared Mahalanobis distance from the mean using the chi-squared distribution on 2 degrees of freedom.

### Reporting summary

Further information on research design is available in the Nature Research Reporting Summary linked to this paper.

## Online content

Any methods, additional references, Nature Research reporting summaries, source data, extended data, supplementary information, acknowledgements, peer review information; details of author contributions and competing interests; and statements of data and code availability are available at 10.1038/s41586-022-04861-4.

## Supplementary information

Supplementary Video 1 Reference video of a typical swooping perching trajectory. Reference video showing two flights at perch spacing of 12 m by juvenile bird Drogon. In this sequence, the bird is seen wearing a falconry backpack, a falconry tail mount and a falconry hood that had been modified to avoid obscuring the bird’s vision. Retroreflective markers of various sizes are visible attached to the backpack, hood, tail mount and wing feathers, but only the 6.4-mm-diameter backpack markers were tracked for the purposes of this study. The video images have been undistorted automatically using the camera calibration from the motion capture system and the falconer’s face has been anonymized, but no other image manipulations have been applied. Backpack marker paths tracked retrospectively by Vicon Nexus software are overlaid ahead of the trajectories that the markers follow.

Reporting Summary

## Data Availability

The motion capture data that support the findings of this study are available in figshare with the identifier 10.6084/m9.figshare.16529328.

## Code availability

Custom algorithms and a published summary of the analysis code are available in figshare with the identifier 10.6084/m9.figshare.16529328.
