# Supplementary material for: Optimization of avian perching manoeuvres
Source: Nature. 2022 Jun 29;607(7917):91–6. doi: 10.1038/s41586-022-04861-4 (PMC9259480; doi:10.1038/s41586-022-04861-4)
Supplement: Supplementary file 2 — Reporting Summary [file 41586_2022_4861_MOESM2_ESM.pdf]

nature portfolio

Corresponding author(s): Graham Taylor
Last updated by author(s): Mar 28, 2022

Reporting Summary

Nature Portfolio wishes to improve the reproducibility of the work that we publish. This form provides structure for consistency and transparency in reporting. For further information on Nature Portfolio policies, see our Editorial Policies and the Editorial Policy Checklist.

Statistics

For all statistical analyses, confirm that the following items are present in the figure legend, table legend, main text, or Methods section.

n/a	Confirmed	
☐	☒	The exact sample size (n) for each experimental group/condition, given as a discrete number and unit of measurement
☐	☒	A statement on whether measurements were taken from distinct samples or whether the same sample was measured repeatedly
☐	☒	The statistical test(s) used AND whether they are one- or two-sided<br>*Only common tests should be described solely by name; describe more complex techniques in the Methods section.*
☐	☒	A description of all covariates tested
☐	☒	A description of any assumptions or corrections, such as tests of normality and adjustment for multiple comparisons
☐	☒	A full description of the statistical parameters including central tendency (e.g. means) or other basic estimates (e.g. regression coefficient) AND variation (e.g. standard deviation) or associated estimates of uncertainty (e.g. confidence intervals)
☐	☒	For null hypothesis testing, the test statistic (e.g. F, t, r) with confidence intervals, effect sizes, degrees of freedom and P value noted<br>*Give P values as exact values whenever suitable.*
☒	☐	For Bayesian analysis, information on the choice of priors and Markov chain Monte Carlo settings
☒	☐	For hierarchical and complex designs, identification of the appropriate level for tests and full reporting of outcomes
☒	☐	Estimates of effect sizes (e.g. Cohen's d, Pearson's r), indicating how they were calculated

Our web collection on statistics for biologists contains articles on many of the points above.

Software and code

Policy information about availability of computer code

Data collection	Motion capture data were collected using Vicon Nexus 2 software (Vicon Motion Systems Ltd, Oxford, UK).
Data analysis	Motion capture data were reconstructed using Vicon Nexus 2.7.6 software (Vicon Motion Systems Ltd, Oxford, UK). Motion capture data were processed in Matlab 2018a (The Mathworks Inc., Natick, MA). Flight dynamics modelling and statistical analysis were performed in Matlab 2020a. Custom algorithms and a published summary of the analysis code are available in figshare with the identifier doi:10.6084/m9.figshare.16529328.

For manuscripts utilizing custom algorithms or software that are central to the research but not yet described in published literature, software must be made available to editors and reviewers. We strongly encourage code deposition in a community repository (e.g. GitHub). See the Nature Portfolio guidelines for submitting code & software for further information.

Data

Policy information about availability of data

All manuscripts must include a data availability statement. This statement should provide the following information, where applicable:

- Accession codes, unique identifiers, or web links for publicly available datasets
- A description of any restrictions on data availability
- For clinical datasets or third party data, please ensure that the statement adheres to our policy

The motion capture data that support the findings of this study are available in figshare with the identifier doi:10.6084/m9.figshare.16529328

Field-specific reporting

Please select the one below that is the best fit for your research. If you are not sure, read the appropriate sections before making your selection.

☒ Life sciences ☐ Behavioural & social sciences ☐ Ecological, evolutionary & environmental sciences

For a reference copy of the document with all sections, see nature.com/documents/nr-reporting-summary-flat.pdf

Life sciences study design

All studies must disclose on these points even when the disclosure is negative.

Sample size	As is typical in animal biomechanics, the scientific inferences contained in this manuscript are based on a detailed analysis of individual dynamics, where individual morphological variation is contained explicitly within the dynamics modeling. A sample of n=4 Harris' Hawks (Parabuteo unicinctus) was chosen on grounds of practicality, and on the basis that this was expected to be sufficient to confirm the repeatability of the findings on individual dynamics. Each individual was sampled repeatedly under four experimental conditions to establish the consistency of the individual dynamics within each test condition, and the total sample of 1,585 flights was designed to be substantially higher than in other comparable studies of avian flight.
Data exclusions	We excluded 9 out of 1,585 flights for which the lowest point in the bird's trajectory deviated by >3 standard deviations from the marginal mean for that individual and test condition in a linear mixed effects model. Post hoc investigation of these trajectories confirmed that two were attributable to measurement error arising from tracking errors in the motion capture system; the remainder comprised atypical trajectories, and/or flights for which the lowest point in the bird's trajectory did not provide a good estimate of the location of the transition from powered to gliding flight. The latter occurred in cases where the transition from powered to gliding flight happened at a local, rather than global, minimum in height. The number of outliers is a small percentage of the total number of flights (0.6%), and the results of the statistical analysis were qualitatively the same whether they were included or excluded. Nevertheless, because these outliers do not accurately characterise the behaviour that the flight dynamics model describes, we consider it better to exclude them as noise.
Replication	Subsequent research using the same n=4 individuals and one additional individual have displayed the same flight behaviour in each of four further years, although no attempt has been made to quantify the extent of any small changes to the swooping trajectories observed over this period.
Randomization	All individuals were tested under all experimental conditions.
Blinding	Blinding the experimenters to the test condition was not possible because the experiment involved flying each bird between two perches at a fixed spacing distance that was varied experimentally. The identity of the bird and the spacing of the perches was therefore known to the experimenters, and is implicit in the resulting data structure, but the physical nature of the measurements means that this is unlikely to have biased the results.

Reporting for specific materials, systems and methods

We require information from authors about some types of materials, experimental systems and methods used in many studies. Here, indicate whether each material, system or method listed is relevant to your study. If you are not sure if a list item applies to your research, read the appropriate section before selecting a response.

Materials & experimental systems

n/a	Involved in the study	
☒	☐	Antibodies
☒	☐	Eukaryotic cell lines
☒	☐	Palaeontology and archaeology
☐	☒	Animals and other organisms
☒	☐	Human research participants
☒	☐	Clinical data
☒	☐	Dual use research of concern

Methods

n/a	Involved in the study	
☒	☐	ChIP-seq
☒	☐	Flow cytometry
☒	☐	MRI-based neuroimaging

Animals and other organisms

Policy information about studies involving animals; ARRIVE guidelines recommended for reporting animal research

Laboratory animals	Captive-bred Harris' Hawks (Parabuteo unicinctus), comprising three juvenile males (<0.5 years) and one adult female (7 years) .
Wild animals	No wild animals were used in this study.
Field-collected samples	No field-collected samples were used in this study.

Ethics oversight	This work was approved by the Animal Welfare and Ethical Review Board of the Department of Zoology, University of Oxford, in accordance with University policy on the use of protected animals for scientific research, permit no. APA/1/5/ZOO/NASPA, and is considered not to pose any significant risk of causing pain, suffering, damage or lasting harm to the animals. The research was also subject to ethical review by the European Research Council (ERC) Executive Agency, with which detailed protocols are deposited.

Note that full information on the approval of the study protocol must also be provided in the manuscript.
